# Hydrogel Layers on the Surface of Polyester-Based Materials for Improvement of Their Biointeractions and Controlled Release of Proteins

**DOI:** 10.3390/polym8120418

**Published:** 2016-12-02

**Authors:** Viktor Korzhikov-Vlakh, Maria Krylova, Ekaterina Sinitsyna, Elena Ivankova, Ilia Averianov, Tatiana B. Tennikova

**Affiliations:** 1 Institute of Chemistry, St. Petersburg State University, Universitetskii Pr. 26, Peterhoff, 198504 St. Petersburg, Russia; v_korzhikov@mail.ru (V.K.-V.); new-masika@mail.ru (M.K.); 2 Institute of Macromolecular Compounds, Russian Academy of Sciences, Bolshoy Pr. 31, 199004 St. Petersburg, Russia; kat_sinitsyna@mail.ru (E.S.); ivelen@mail.ru (E.I.); averianovilia@gmail.com (I.A.)

**Keywords:** poly(lactic acid), surface modification, hydrogel, protein adsorption, protein release, cell-material interactions

## Abstract

The modification of bioresorbable polyester surfaces in order to alter their biointeractions presents an important problem in biomedical polymer science. In this study, the covalent modification of the surface of poly(lactic acid)-based (PLA-based) films with poly(acryl amide) and sodium alginate hydrogels was performed to change the non-specific polyester interaction with proteins and cells, as well as to make possible the covalent attachment of low-molecular weight ligands and to control protein release. The effect of such modification on the film surface properties was studied. Parameters such as swelling, water contact angle, surface area, and binding capacity of low-molecular weight substances were evaluated and compared. The comparative study of adsorption of model protein (BSA) on the surface of non-modified and modified films was investigated and the protein release was evaluated. Cell viability on the surface of hydrogel-coated films was also tested. The developed approach could be applied for the modification of PLA-based scaffolds for tissue engineering and will be further studied for molecular-imprinting of biomolecules on the surface of polyester-based materials for control of biointeractions.

## 1. Introduction

Relatively bioinert resorbable polyesters, such as poly(lactic acid) (PLA), poly(lactic-*co*-glycolic acid) (PLGA), and poly(caprolactone) (PCL) have found numerous applications in various biomedical applications. Among them, the formation of scaffolds for tissue engineering [1,2,3,4] and particles for drug delivery systems [4,5,6,7,8] are considered as the leading applications. However, there are several inherent drawbacks of the polyester-based material surface, which limit the use of these materials. These are the significant hydrophobicity [9,10,11,12,13], which results in non-specific interactions with cells and surrounding tissues [1,13,14,15], as well as poor chemical functionality [4,14,16] that prevents the biofunctionalization of the materials. The modification of PLA-based material surface properties was shown to be crucial for their interaction with cells and tissues and respectively their biomedical applications [1,12,16,17].

Numerous attempts to modify the surface of biomedical polyester were performed and described in [16]. They included the non-covalent and covalent attachment of functional biomolecules. The non-covalent methods are represented by: protein [18] or polymer [19,20,21] adsorption; entrapment of hydrophilic natural [22] or synthetic [23,24] polymers using reversible swelling of the PLA surface; application of special migratory amphiphilic macromolecules to the bulk PLA, which contain bioligands (such as RGD-peptides) and adsorb on the surface [25,26]; and plasma treatment [27,28] that leads to increasing the surface wettability and enhanced cell attachment. The covalent PLA modification is possible via: wet chemistry, such as surface hydrolysis with subsequent activation of carboxylic groups with carbodiimide [29]; and liquid [30] or vapor [31] photografting of functional monomers to introduce reactive groups on the surface. Some of cited studies just aimed to increase the hydrophilicity of the PLA-surface, while the others used strategies of special bioligand attachment. In terms of biofunctionalization, which is aimed at regulating cell-material interaction, the latter approach allows the introduction of specific biomolecules with cell-instructive properties, which is more advantageous for success in tissue engineering applications [2]. Moreover, some bioligands, such as RGD-peptides and other adhesion factors, need to be stable when attached to the surface, while the other motifs, such as growth factors, could be released from the surface. Thus, it seems to be interesting to form a special hydrogel on the surface of PLA, which will be able to both covalently attach the peptides and to controllably release the proteins.

The surface-located hydrogels are of significant interest due to the possibility to increase the functionality of biomaterials. Polysaccharide hydrogels were shown to be suitable candidates for the modification of scaffold supports. In [32], chitosan/hyaluronic acid hydrogel was formed on the surface of PCL fibers and cross-linked with water-soluble carbodiimide. In [33], hyaluronan hydrogels cross-linked with divinyl sulfone were applied to cover the hydrophobic poly(ethylene acrylate) surface. In cited papers, the significant surface hydrophilization, e.g., water uptake, increased and improved protein adsorption and cell viability were reported. In addition, such hydrogel layers could be used for the construction of scaffolds enabling local controlled protein [33] or drug [34] release. More recently, the alginate-based cell-loaded hydrogel bioink was applied for PCL struts coating [35]. However, such hydrophilic layers were not covalently attached to the hydrophobic support surface and thus their possible detachment could occur. In this respect, synthetic systems that are covalently bound to the surface of a biomaterial matrix seem preferable [36]. Moreover, such synthetic approaches could provide higher control over the hydrophilic/hydrophobic balance, layer thickness, and reactivity towards various bioligands to be attached to the surface of biomaterials [36]. Such hydrogel coatings could be especially interesting for improvement of the surface properties of 3D-printed PLA composite fibers reinforced with microcrystalline cellulose [37]. Nevertheless, to the best of our knowledge, no data on covalently attached hydrogels to PLA surfaces have been published. It is also interesting to compare synthetically formed hydrogels with those obtained from natural polymers.

In the present study, the formation of covalently attached hydrogel coatings on the surface of PLA-based films was performed. The surfaces of such films were modified to introduce the reactive groups. The hydrogel coating was formed using a free-radical copolymerization process, or the cross-linking of natural polymers. The copolymerization of acrylamide with *N*,*N*′-methylene-bis-acrylamide was carried out in former case, while in the latter approach, alginate cross-linking with l-lysine in the presence of water-soluble carbodiimide was performed. The morphology of such coatings, as well as their effects on PLA-based surface properties were studied and compared. Both hydrogel coatings were loaded with model protein (BSA) to study its controlled release.

In further research, the hydrogel coatings will be formed on the surface of supermacroporous PLA-based scaffolds for connective tissue regeneration. Such hydrogels could be used for the improvement of surface cell instructive properties via attachment of special bioligands for cell receptors and local release of tissue-specific growth factors. Moreover, the molecular imprinting of cellular receptors into the structure of such hydrogels could allow us to direct cell-material interactions without the application of expensive proteins. In order to test the viability of cells on the surface of the hydrogels, the Human embryonic lung fibroblasts (HELF) cell line, which resembles connective tissue cells, was applied. In the case of cell adhesion visualization experiments, the cells with potency for differentiation into connective tissue cells, namely, Human mesenchymal stem cells (HMSCs), were used with regard to the future practical application of such supports.

## 2. Materials and Methods

### 2.1. Materials and Instruments

1-hydroxybenzotriazol (HOBT), 1-ethyl-3-(3-dimethylaminopropyl)carbodiimide (EDC), ethylene diamine (EDA), glycidyl methacrylate (GMA), acrylamide (AAm), *N*,*N*′-methylene-bis-acrylamide (MBAAm), ammonium peroxodisulfate (APS), *N*,*N*,*N*′,*N*′-tetramethylethylene-1,2-diamine (TEMED), l-lysine, and 2,4,6-trinitrobenzenesulfonic acid (TNBS) were purchased from Sigma-Aldrich (Darmstadt, Germany) and used without special purification. Sodium alginate with low viscosity (Alg, molecular weight 216 kDa, M:G (61%:39%)) was a product of Alfa Aesar (Heysham, UK). The salts used for the preparation of buffer solutions were products of Fluka (Buchs, Switzerland). Organic solvents applied for polymer synthesis and film preparation, namely toluene and chloroform, were purchased from Merck (Darmstadt, Germany). PLA with *M*w 250,000 was synthesized as described earlier [38,39,40]. BF-6 medical glue (Vertex, St. Petersburg, Russia) was used for the attachment of films to the bottom of 24 well plates before cell viability tests.

All dry reagents were weighted with OHAUS Adventurer Pro balances (Parsippany, NJ, USA). Freeze-drying of gels and films was performed with LABCONCO Freezone 1L equipment (Kansas City, MO, USA). The ^1^H NMR and IR spectra of polymers were measured using a Bruker AV400 (Brucker, Rheinstetten, Germany). A Thermostat (Binder KB 53, Tuttlingen, Germany) was used for solvent evaporation during film preparation. A Heidolph Unimax 1010 rotation shaker (Schwabach, Germany) and Vortex (Biosan, Riga, Latvia) was applied for reaction mixture stirring. Optical densities of solutions were analyzed with a UV-1800 Spectrophotometer (Shimadzu, Kyoto, Japan). The film surface was imaged using a scanning electron microscope Zeiss Supra 55VP (Oberkochen, Germany). The samples were sputter coated with gold-palladium alloy using a sputter coater (Anatech Hummer 6.2, Garfield, NJ, USA). The measurements of contact angles were performed using Drop Shape Analyser (DSA) 100E (Kruss, Hamburg, Germany) equipment running 1.80.0.2 software. AFM measurements were carried out with Research modular platform Nanolab equipped with a UHV chamber with a scanning probe microscope Omicron VT AFM XA 50/500(CEO Scienta Omicron, Uppsala, Sweden), allowing the study of the surface structure with atomic resolution.

### 2.2. Methods

#### 2.2.1. PLA Film Preparation

**Figure 1:**
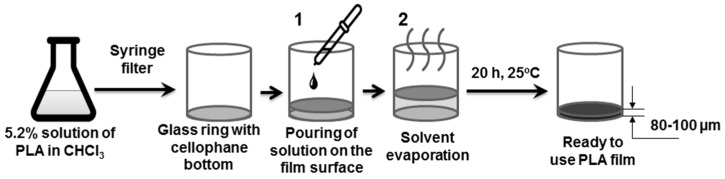
Poly(lactic acid) (PLA-based) film preparation using a glass ring with cellophane bottom: 1—addition of the PLA solution; 2—slow solvent evaporation.

200 mg (5 wt %) of PLA were dissolved in 3.75 g of chloroform during a 24 h period. The prepared solution was filtered with a hydrophobic PTFE syringe filter to get rid of possible particulate impurities and poured onto a cellophane surface, fixed on a glass ring with a 33 mm diameter (Figure 1). The ring was placed on the aligned table inside the thermostat fixed at 30 °C and the chloroform was slowly evaporated during a 24 h period, resulting in the formation of a thin film. Further modifications of the film surface were carried out according to the described experimental design, namely, without taking the film off the ring.

#### 2.2.2. Preparation of Hydrogel Coated PLA-Based Films

Two types of hydrogel formation approaches, namely, polyacrylamide and alginate, started with partial saponification of ester groups on the surface of the PLA-based film. For that, the films were treated with 10 mL of 0.1 M NaOH for 30 min. To stop the process of polyester hydrolysis, the films were washed 4 times with 10 mL of distilled water for 10 min.

##### Formation of Poly(acrylamide) (PAAm) Gel on the Surface of PLA Films

**Figure 2:**
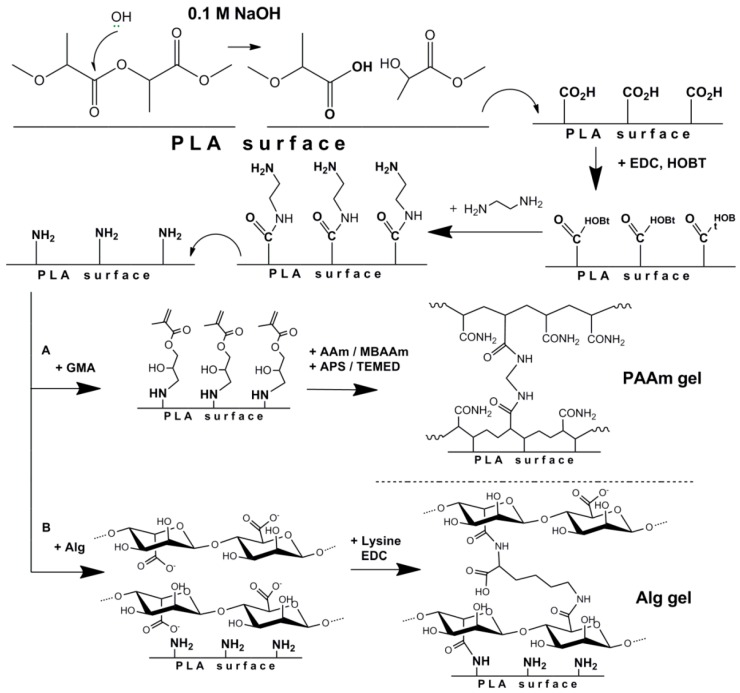
Scheme of the modification of PLA and formation of hydrogel films using two approaches: (**A**) copolymerization of acrylamide with methylene-bis-acrylamide; (**B**) cross-linking of alginate with lysine.

Firstly, methacrylate groups were introduced on the surface of fabricated films using the developed multistep procedure (Figure 2). The process includes (1) activation of carboxyl groups; (2) coupling of diamine; and (3) attachment of the compound bearing methacrylic double bond. After each reaction step, the film surface was washed 3 times with distilled H_2_O for 10 min. To activate the COOH-groups, 5 mL of HOBT with a concentration of 1 mg/mL 0.05 M 2-(N-morpholino)ethanesulfonic acid (MES) buffer, pH 5.4, was poured onto the film. The reaction system was slowly cooled down to 10 °C during 5 min. Then 5 mL of EDC with a concentration of 1 mg/mL 0.05 M MES-buffer, pH 5.4, was added. The formation reaction of active ester proceeded for 50 min at 5 °C with permanent stirring at 70 rpm. After this step, the surface was modified with EDA; 50 µL (45 µg) of EDA in 10 mL of 0.01 M phosphate buffer saline (PBS, pH 7.4) was poured on the surface (reaction time—30 min, temperature—25 °C). Then 100 µL of GMA dissolved in 10 mL 0.01 M PBS, pH 7.4, was poured onto the surface of the PLA film (reaction time—20 h, temperature—25 °C).

AAm polymerization on the surface of PLA-based films was typically performed as follows; 45 mg of AAm and 15 mg of MBAAm (AAm/MBAAm = 6 mol/mol) were dissolved in 2 mL of water, and separately, 1 mg of APS was dissolved in 940 µL of water. The monomers and APS were cooled down separately to 5 °C during 5 min in an ice bath to prevent the reaction. Furthermore, the monomers were mixed and introduced into a tube containing 1 mg of TEMED and was vortex stirred for 15 s. Finally, 3 mL of the mixture were poured on the surface of the film (reaction time—20 h, temperature—25 °C). The films were washed 6 times with distilled H_2_O for 10 min and dried in air.

##### Formation of Alginate (Alg) Gel on the Surface of PLA Films

Similar to the case of glycidyl methacrylate attachment, the partial saponification of PLA ester groups with NaOH was performed. By action the carboxylic groups were formed on the surface of the films. Further activation of COOH groups with EDC and HOBT was performed similarly to the procedure described above. Then, 10 mg of 1,2-ethylenediamine in 10 mL of sodium borate buffer, pH 8.4, was poured onto the film (time of reaction—30 min, temperature—25 °C, (Figure 2)). As mentioned before, after each step the films were washed 3 times with distilled H_2_O for 10 min.

The cross-linking reaction of Alg on the surface of PLA-based films was typically performed as follows; 1 mL of 1 wt % solution of Alg was diluted by 4 mL of 0.01 M Na-borate buffer solution (BBS), pH 8.4. After that, 5 mL of l-lysine (1 mg/mL) solution was added to the mixture. Then, 1 mL of EDC (1 mg/mL) solution at BBS, pH 8.4 was added to the obtained mixture, and the mixture was vigorously stirred and immediately poured onto the surface of the PLA-film (reaction time—20 h, temperature—25 °C). The films were washed 6 times with distilled H_2_O for 10 min and dried in air.

#### 2.2.3. Materials Characterization

##### Determination of Carboxylic Group Concentration on the PLA-Based Film Surface

The reaction of surface COOH groups with glycine was performed. For that, the groups were activated by EDC and HOBt following the procedure described above. The difference of the glycine amount in the solution before and after the coupling reaction was determined using the ninhydrin reaction [41] and a previously plotted calibration curve.

The procedure was generally performed as follows; 3 mL of 0.1 mg/mL glycine solution in buffered sodium borate, pH 9.4, was poured onto the surface of the PLA-based film, which was previously saponified and activated with EDC/HOBt. After the reaction proceeded, 0.5 mL of reaction solution was taken for analysis. For that, the probe was placed in a 15 mL plastic tube provided with a cap. Then 2.5 mL of 0.2 wt % ninhydrin solution in ethanol was added. The cap was tightly closed and the tube was heated to 120 °С for 40 min using a glycerin bath. Then the tube was cooled down and the optical density of the colored solution was measured at λ = 400 nm. The calibration curve was plotted with the application of glycine solutions of known concentration.

The quantity of carboxylic groups on the surface of PLA-based films was calculated as follows:

(1) $$q\left(\mathrm{COOH}\right)=\frac{C_{\mathrm{before}}\left(\mathrm{Gly}\right)-C_{\mathrm{after}}\left(\mathrm{Gly}\right)}{V\times M\left(\mathrm{Gly}\right)},$$

*С*_before_ and *С*_after_—the concentration of glycine in a solution before and after the reaction with activated carboxylic groups on the surface (*C = D*_400_*/*ε, *D*—optical density, ε—coefficient proportional to extinction); *V*—solution volume (3 mL); *M*(Gly)—molecular weight of glycine.

##### Determination of Amino Groups Concentration on the Surface of PLA-Based Film Surface

The films were destroyed by treating with 1 M NaOH for 4 h at 45 °C. In order to estimate the primary amine amount, the pH of solution was adjusted to 9.4 and the reaction of amino groups with TNBS was performed. For that, 1.5 mL of BBS, pH 9.4, and 0.1 mL (8 mg/mL) of TNBS were added to a 0.5 mL aliquot. The reaction was allowed to proceed for 50 min. Then the reaction was stopped by adding 1.5 mL of acetic buffered solution, pH 4.8. The optical density of the obtained solution, caused by absorption of primary amine-TNBS adduct at λ = 420 nm was measured. The amount of amino groups was determined using a previously plotted calibration curve with cystamine as a standard.

##### Film Thickness

**Figure 3:**
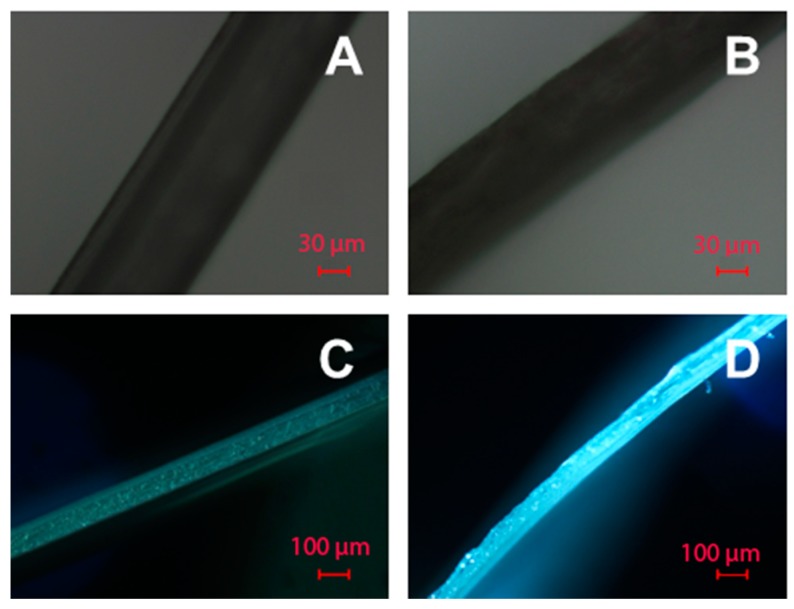
The images of the cuts of modified PLA films obtained by optical microscopy: (**A**,**B**) PLA-PAAm and PLA-Alg in transmitted light; (**C**,**D**) PLA-PAAm and PLA-Alg in reflected light.

The thickness of dry films and hydrogel layers was evaluated by optical microscopy (Nikon Eclipse E200, Tokyo, Japan). The films were gently cut and analyzed in reflected and transmitted light (Figure 3). The measurements were performed using a 10× magnification objective lens. The microphotographs obtained were examined with the free open source ImageJ software (National Institutes of Health, Bethesda, MD, USA). Ten measurements were made for each film sample, and the average thickness values are presented in Table 1.

##### Swelling

The films obtained were dried in vacuum and weighed (*m*_dry_). Then the samples were placed in 15 mL of 0.01 M PBS (pH 7.4) and incubated at room temperature for 3 days. After that, the films were removed from solution. The excess liquid was carefully removed from the film surface and the films were weighed again (*m*_wet_). To calculate the swelling values (*N*), the following formula was used:

(2) $$N=\frac{m_{\mathrm{wet}}-m_{\mathrm{dry}}}{m_{\mathrm{dry}}}\times 100\%,$$

##### Scanning Electron Microscopy (SEM)

The samples were sputter coated with gold-palladium alloy and subsequently viewed at accelerating voltages of 5–15 kV.

##### Contact Angle

Routinely, a 1.5 μL water drop was placed on the film surface, and the average static contact angle was determined using the drop analysis plugin (Biomedical Imaging Group, Lausanne, Switzerland) for ImageJ software during the first 30 s following the drop deposition. For each sample, ten measurements were done on different areas of the film. Three samples were characterized for each film composition.

##### Atomic Force Microscopy (AFM)

The films were dried before measurement and the sheets with area 1 cm^2^ were cut out. The scanning area 5 µm × 5 µm was applied. The free open source WxSM ver. 5.0 develop 8.2 software (Nanotec, Madrid, Spain) was used for data processing. The real swept surface area was estimated from AFM data. The ratio of geometrical surface (*S*_0_) to the real surface (*S*_r_) was determined to characterize the roughness of the modified material. *S*_0_ was calculated as the product of the measured square sides (typically 5 µm × 5 µm). *S*_r_ was estimated by using the above mentioned software.

#### 2.2.4. Protein Adsorption

The study of the kinetics of adsorption of the model protein (BSA), as well as the building of adsorption isotherms, were performed at static conditions in 0.01 M sodium phosphate buffer, pH 7.4. The determination of the protein in a supernatant solution was carried out colorimetrically using a Lowry–Folin assay [42]. The optical density of colored solutions was measured with a Shimadzu UV-1800 spectrophotometer. The masses of the film sheets were 8–10 mg. The amount of adsorbed protein (*Q*_ads_) was calculated according to the following equation:

(3) $$Q_{\mathrm{ads}}=\frac{q_{\mathrm{ads}}}{m_{\mathrm{film}}}=\frac{q_{0}-q_{i}}{m_{\mathrm{film}}}=\frac{V_{0}C_{0}-V_{i}C_{i}}{m_{\mathrm{film}}},$$

where *q*_ads_ is the amount of the adsorbed protein, which is equal to the loss of protein quantity in the solution before (*q*_0_) and after (*q_i_*) adsorption; *V*_0_, *V_i_* and *C*_0_, *C_i_* are respectively the volumes and the concentrations of the initial solution and the solution after adsorption, respectively. The concentration was evaluated from the optical density with the application of a previously plotted calibration curve.

##### Kinetic Studies

BSA solution with a concentration 1 mg/mL was prepared in 0.01 M PBS (pH 7.4). Each studied film was placed in a separate Eppendorf microtube and filled with 2 mL of BSA solution. The measurements of a change of protein concentration were carried out during 3 h every 15 min from the moment of the immersion of a film into BSA solution. The protein concentration in the supernatant was then measured after 6, 12, and 24 h.

##### Isotherm Building

The masses of chosen PLA, PLA-PAAm, and PLA-Alg film sheets were 3–5 mg. Protein solutions with the following concentrations were prepared: 3.0; 2.0; 1.0; 0.8; 0.5; 0.2 and 0.1 mg/mL. Then the solutions were incubated with the pre-weighed films for 1 to 7 days.

#### 2.2.5. Study of Protein Loading and Controlled Release

Bovine serum albumin (BSA) was used as a protein model to evaluate the potential of the surface film coatings as a controlled release system. The loading of BSA into PAAm and Alg gels was performed using different approaches.

In the case of PAAm gel, 5 mg of BSA was dissolved in 0.5 mL of water and added to the mixture of monomers before polymerization. The rest of the polymerization procedure was performed as described earlier (see the section above).

To load the Alg gel with BSA, the swelling-diffusion approach was applied. For that, the PLA films with Alg gel on the surface were swollen in sodium borate buffer, pH 9.0, for 2 h. Then 5 mL of BSA solution in PBS, pH 9.0, with 1 mg/mL concentration, was added and stirred overnight at 37 °C.

After protein loading, the films were washed once with water and filled with 1.0 mL of fresh PBS, pH 7.4, to distinguish the protein release. The supernatants were collected at selected time points and replaced with fresh PBS, pH 7.4. The release was performed in an incubator, which was thermostated at 37 °C. The BSA concentration in each supernatant was established by the Lowry-Folin assay as mentioned above. The amount of desorbed protein was determined during 1 day and after 7 days. The protein desorption was quantified according to the equation below:

(4) $$Q_{\mathrm{released}}=\frac{q_{\mathrm{released}}}{m_{\mathrm{film}}}=\frac{C_{\mathrm{protein}}\;V_{\mathrm{solution}}}{m_{\mathrm{film}}},$$

where *q*_released_ is the quantity of BSA in a supernatant, which is determined as a product of protein concentration in a supernatant and its volume; *m*_film_ is the mass of the film sheet used for the release study.

#### 2.2.6. Hydrolysis of PLA-PAAm

Warm (35–45 °C) 0.1 M NaOH solution was poured onto the surface of a PLA-PAAm film. The reaction was carried out for 30 min with slight stirring at 40 °C. The hydrolyzed film surface was washed three times with distilled water and used for further reactions.

#### 2.2.7. Modification of Hydrogel Surface with Glycine

PLA-Alg and hydrolyzed PLA-PAAm films were modified with glycine according to the procedure described above (see the section Determination of surface carboxylic groups).

#### 2.2.8. Cell Culture Experiments

##### Cells

Human embryonic lung fibroblasts (HELF) were purchased from Biolot (St. Petersburg, Russia). Human mesenchymal stem cells (HMSCs) were derived from fat tissue (huF 62) [43]. Both cell types were cultivated into Dulbeccos’ Modified Eagle Medium containing 10% fetal calf serum, 1% penicillin/streptomycin, and 1% l-glutamine.

##### Evaluation of Cell Viability on the Surface of Modified Films

The films were cut to the round shaped sheets and attached to the bottom of 24-well plates by medical glue (BF-6, polyvinylbutyral based adhesive). 5000 cells per well were seeded on every film and were adhered for 30 min at 37 °C/5% CO_2_ with slight stirring. Afterwards, the wells were filled with cell culture medium. After 1 and 24 h, the cell viability was evaluated by using a 3-(4,5-dimethylthiazol-2-yl)-2,5-diphenyltetrazolium bromide (MTT) assay, which was performed with *n* = 7 and control cells incubated on the surface of the plate itself. Cells/films were incubated with 100 μL of fresh cell culture medium and 10 μL MTT (5 mg/mL in PBS) for 4 h at 37 °C/5% CO_2_. Formazan crystals were dissolved overnight with 10% sodium dodecyl sulfate (SDS) in 0.01 M HCl. The formazan absorption was measured at 570 nm subtracted from the 630 nm absorption. The relative cell viability (%) was expressed as a percentage relative to the control cells cultured directly on the well surface coated with glue.

##### DAPI-Staining

HMSCs were seeded on the film surface similar to the procedure described above. The cells were incubated for 24 h before treatment with the dye. For the staining procedure, four replicates of each modified film and the untreated PLA film were carried over into a new 24-well plate and washed twice with PBS. The cells adhered on the matrices were fixed with ice-cold ethanol for at least 20 min. After the fixation, they were washed twice with PBS, pH 7.4, and then incubated in DAPI solution for 15 min at 37 °C. Fluorescence microscopy was performed after washing the films twice with PBS, pH 7.4, by using a Leica DM1000 LED (Wetzlar, Germany) at 460/360 nm.

##### Statistical Analysis

All values were given as means and standard error of a mean value (SEM) was calculated. A normality test and a test for equal variances were performed before running a Student’s two-tailed *t*-test to compare results between two groups. In the case of unsatisfied correlation of the data, the normality Mann-Whitney-Rank-Sum test was performed. A *p*-value < 0.05 was considered as statistically significant.

## 3. Results and Discussion

### 3.1. PLA Film Formation and Hydrogel

The aim of the present research was to study the formation of covalently attached hydrogel layers on the surface of poly(lactic acid) (PLA) films, which, first, hydrophilize the surface and, second, allow for the incorporation of biomacromolecules. Such layers can be very interesting regarding the improvement of surface interactions with proteins from bioliquids and cells, as well as being useful for the formation of systems providing the controlled release of molecules of interest.

For preparation of the PLA-based films, the dissolution-evaporation method was developed and applied (Figure 1). A polymer of high molecular weight was synthesized according to the previously published procedure [40]. The high molecular weight was needed for preparation of self-supported films. The comparison of poly(l-lactic acid) (PLLA) and poly(d,l-lactic acid) (PDLLA) showed that the films based on PLLA were more brittle, while the second polymer yielded elastic films, which were much handier for the current research. Thus, in further discussions only PLA notification will be used.

It appeared to be impossible to form the hydrogels directly on the surface of the initial PLA because of the instability of the systems obtained. The hydrogel layer exfoliated from the PLA surface after 2 h storage in 0.01 M PBS, pH 7.4. The introduction of the reactive groups to the surface structure of PLA-based films seemed to be reasonable to provide the covalent attachment of a hydrogel layer.

**Figure 4:**
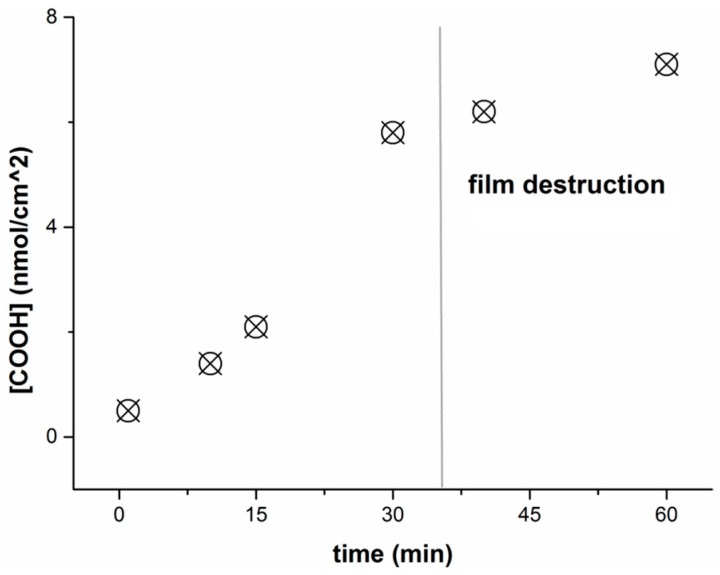
Effect of the time of treatment of the PLA film with 0.1 M NaOH on the surface concentration of generated carboxylic groups.

Firstly, the PLA films were modified using controlled saponification with 0.1 M NaOH in order to generate the carboxylic groups. The concentration of the generated groups was evaluated via the coupling of low molecular weight glycine. The effect of film treatment with NaOH, namely, the dependence of the amount of introduced carboxyls on the time of treatment, was studied (Figure 4). Obviously, the quantity of COOH-groups increases with increasing reaction time. It should be noted that the films stayed stable during 30 min of treatment. Longer treatment with sodium hydroxide led to the destruction of a film into the separated pieces. A 30 min reaction resulted in the maximum amount of carboxylic groups with permanent stability of the film.

The second step of the modification process was the reaction of generated COOH-groups with 1,2-ethylenediamine. For that, they were converted into the active ester by the reaction with HOBt in the presence of EDC followed by the addition of diamine excess (Figure 2). The amount of amino groups introduced onto 1 cm^2^ of the film surface was established after the destruction of the films with 1.0 M NaOH followed by the reaction with TNBS and the detection of a fluorescence adduct. The quantity of the introduced amine was equal to 4.1 nmol/cm^2^.

**Figure 5:**
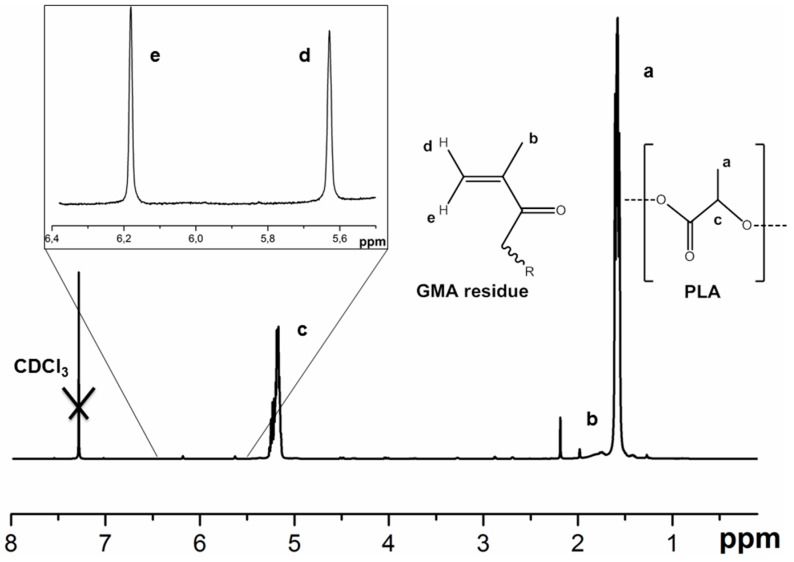
^1^H NMR of the PLA film modified with glycidyl methacrylate GMA and dissolved in CDCl_3_. Both signals of PLA (**a**,**c**) and GMA residue (**b**,**d**,**e**) can be observed.

Two different approaches were applied to obtain spatially cross-linked layers of hydrogel on the surface of aminated PLA materials. The first is the free-radical copolymerization of acrylamide (AAm) with *N*,*N*′-methylene-bis-acrylamide (MBAAm), resulting in the formation of polyacrylamide gel (PAAm gel, Figure 2A). This type of hydrogel represents a well-studied system and allows for protein incorporation at the polymerization step. Such a system is totally synthetic and hard to be degraded. The covalent attachment of the PAAm gel on the surface of the PLA film was provided by the immobilization of glycidyl methacrylate (GMA) on the aminated PLA surface before the polymerization step (Figure 2A). The signals of the introduced methacrylate groups, as well as those of PLA, were detected qualitatively by ^1^H NMR (Figure 5).

To obtain a more biocompatible hydrogel coating, the second approach was used, namely, sodium alginate was cross-linked by l-lysine in the presence of water-soluble carbodiimide (EDC). In this case, alginate gel (Alg, Figure 2B) has been formed on the PLA surface. The covalent attachment of Alg gel to the aminated PLA surface occurs due to the reaction of alginate carboxyl groups with surface amino groups of modified films. The protein loading was carried out using the swelling-diffusion method.

### 3.2. Gel Film Thickness and Swelling

The formation of the gel layer on the PLA surface affects the film thickness, which generally depends on the monomer or polymer concentration taken for the gel formation, as well as on the concentration of the cross-linker (Table 1). The ability to swell compared to the initial PLA layer (sample 1) was found to be a property of all studied films (samples 2–11). It was shown that increasing the monomer (samples 2–5) or polymer (samples 7–9) concentrations led to the growth of the gel layer thickness and more effective swelling of the films due to higher water uptake.

Both in the case of AAm (samples 3, 5, 6) and Alg (samples 8, 10, 11) gels, it was observed that increasing the cross-linker concentration led to slightly thicker films and growth of swelling. The latter can be explained by better water retention in higher cross-linked polymer networks. It should be noted that the use of the highest cross-linker concentration in the case of AAm gels led to the decrease of the gel layer thickness (sample 6). This might happen due to network compaction.

### 3.3. Film Wettability

**Figure 6:**
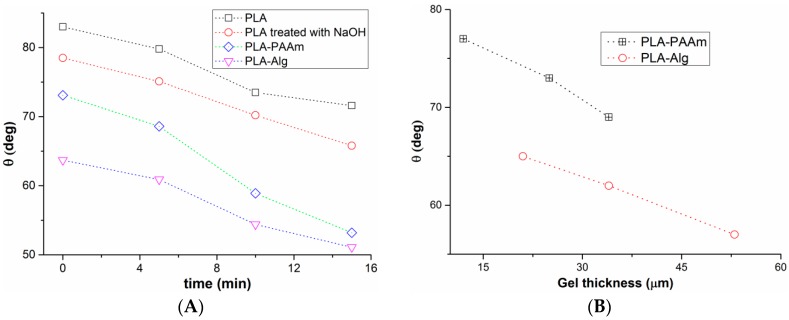
Dependences of the contact angle values measured on the modified films: (**A**) on time; (**B**) on gel thickness.

The contact angles of the water drops on the surface of the initial and modified films were studied to evaluate the effect of modification on the wettability of films. The contact angles of water on the surface of PLA films were plotted as a function of time (Figure 6A) and gel layer thickness (Figure 6B). It was found that the value of the contact angle decreased with the time for all studied films. The contact angles for the PLA film treated with 0.1 M NaOH was lower than that measured for the initial film. The films covered with gel on the surface exhibited a higher extent of a contact angle decreasing with time in comparison with the non-modified PLA layers. The increasing thickness of the gel layer invariably led to wider drop spreading. The dependences obtained can be definitely explained by swelling of the surface gel layer.

### 3.4. The Morphology of PAAm and Alg Gels

The surface of the modified films was analyzed by optical (in reflected and transmitted light), scanning electron and atomic force microscopies. These methods allow the observation of film morphologies at different scales.

**Figure 7:**
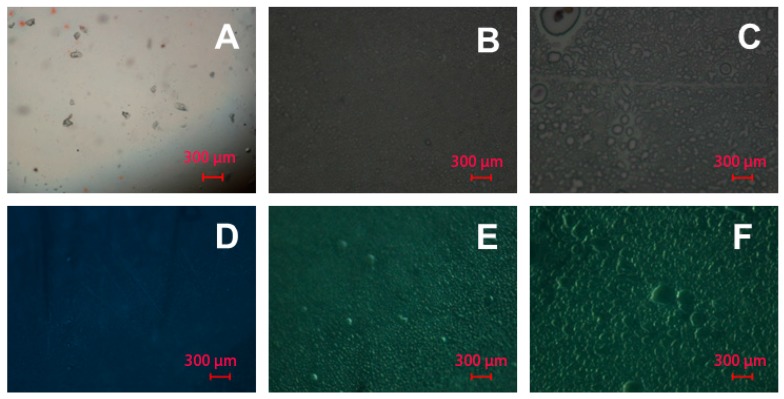
The images of the dried initial and modified PLA surfaces obtained by optical microscopy: (**A**–**C**) PLA, PLA-PAAm, and PLA-Alg in transmitted light, respectively; (**D**–**F**) PLA, PLA-PAAm, and PLA-Alg in reflected light, respectively.

The data obtained by optical microscopy showed that the modification of the surface of PLA films (Figure 7A,D) both by PAAm (Figure 7B,E) and Alg (Figure 7C,F) gels changed their morphology; namely, the surface became more grainy. This can be observed both in reflected (Figure 7B,C) and transmitted light (Figure 7E,F). This observation allows the conclusion that on a micron scale the gels possess grainy structure. The size of the grains appears to be greater in the case of Alg gels. The average grain diameter for the Alg gel was 32 µm versus 14 µm for the PAAm gel.

**Figure 8:**
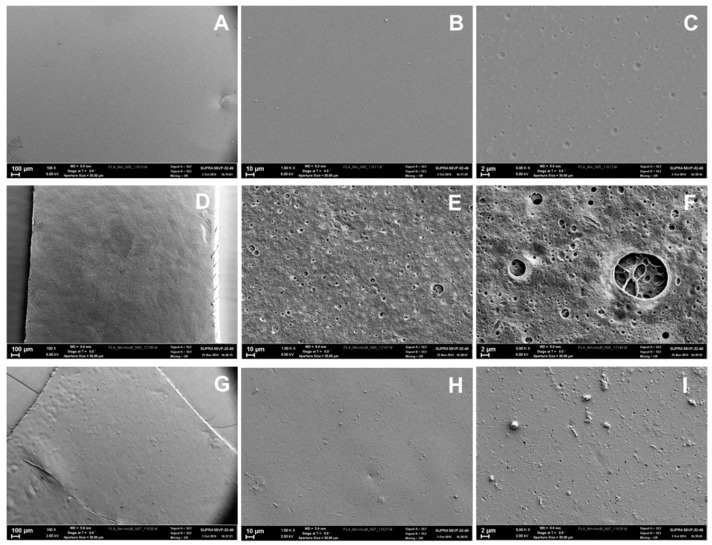
SEM images of the initial and modified surface of the PLA films: (**A**–**C**) initial PLA film; (**D**–**F**) PLA-PAAm; (**G**–**I**) PLA-Alg. The magnification of images in the series is 100×, 1000× and 5000×, correspondingly.

SEM images of the initial and modified PLA films at different magnifications are presented in Figure 8. The images indicate obvious differences in surface morphologies. Similar to the data obtained by optical microscopy, the initial PLA films seem to be quite smooth (Figure 8A,B). Only at 5000× magnification (Figure 8C) can the indentations of 0.5–1.5 µm diameters be observed.

The modified films possess different structures depending on the gel type. PAAm gels (Figure 8D–F) demonstrate numerous holes in their surface, which appear to be interconnected “pores” upon magnification (Figure 8F). In contrast, the Alg gels exhibit a quite homogeneous structure (Figure 8G,H) possessing only small holes with diameters less than 400 nm and aggregate projections about 1 µm in size (Figure 8I). Such variations in gel morphologies can be related to the differences in cross-linking degree that affects gel behavior upon drying. The macromolecules in more highly cross-linked PAAm gel have less freedom for realignment during drying. Thus, the segregation of gel structure occurs followed by the formation of the material with the regions enriched with polymer molecules, while the others represent the pores. In the case of less cross-linked Alg gels, the realignment of macromolecules upon drying is possible and gives a more homogeneous structure.

**Figure 9:**
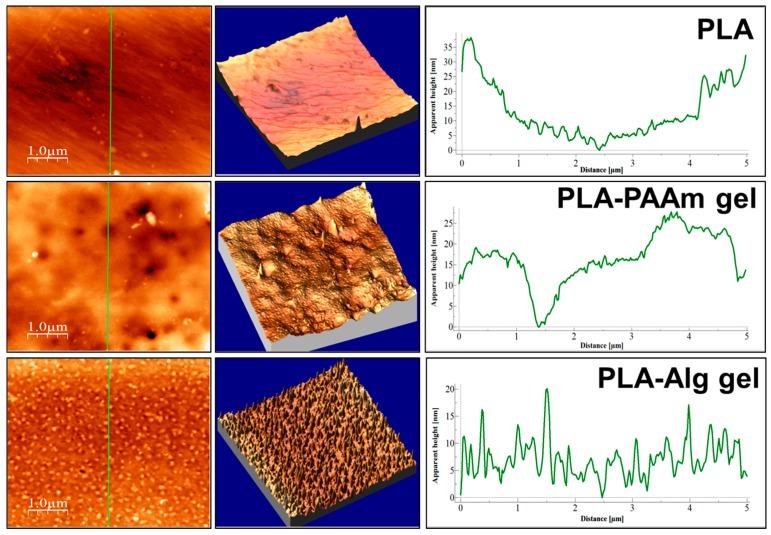
AFM microimages (image size 5 × 5 µm) and surface profiles of the initial and modified gels.

The surface roughness is a very important parameter affecting the interaction with proteins and especially with cells. In order to study the variation of this parameter within the initial and modified films, AFM investigations were performed. The visualization of the film surfaces on a submicron scale (Figure 9) has revealed different topologies of the materials obtained. Despite the fact that all the samples possess undulations, their order greatly differs. For initial PLA films, the period of undulation, which was determined as the distance between two peaks, was equal to 5 µm. Thus, these films are smooth not only on the micron, but also on the submicron scale.

The inspection of images obtained for the PLA-PAAm films allows for observation of the sponge like pattern of the surface. The undulation period was found to be 0.5–1.0 µm. The surfaces of PLA-PAAm films includes projections with diameters of 0.2–1.5 µm and quite deep holes with diameters of 0.5–2.7 µm (Figure 9), which could be attributed to the upper part of the pores as visualized by SEM (Figure 8F).

The AFM microimages of PLA-Alg films (Figure 9) revealed that this type of modification yields the roughest surface. The undulation period was found to be in the range of 30 to 100 nm. The surface of Alg gel has no visible holes but possesses a multitude of sharp projections which are homogenously distributed on the surface.

In order to quantify the roughness of the surface, AFM data were used to calculate the ratio of the geometrical surface (*S*_0_) to the real surface (*S*_r_) (Table 2). *S*_r_, which can be attributed to the real surface of the films, is obviously greater for the modified layers. The Alg gel formation results in more than two times growth of the surface area as compared to the initial PLA film, while the PAAm gel yields much less surface area growth. This result seems to be inconsistent with the SEM data (Figure 8F,I), where the PLA-PAAm films have interconnected pores inside the gel and PLA-Alg gels seem to be quite smooth. It is important to note that the surface areas were calculated using only AFM data, which could not consider the inner surface of the pores. The formation of quite rough, homogenous, sponge like patterns of Alg covering, which gives evident rise to the surface area of the films, is the main result of this study.

### 3.5. Adsorption of Model Protein (BSA)

To examine the ability of the initial and modified films to interact with blood proteins, the adsorption of the model protein (BSA) was studied.

**Figure 10:**
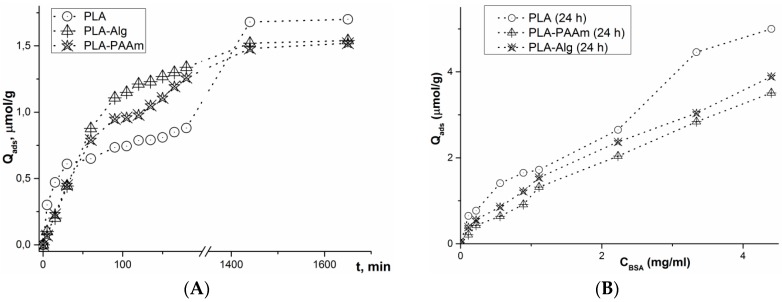
Kinetic curves (**A**) and isotherms (**B**) of BSA adsorption on the surface of the initial and hydrogel modified PLA films.

A comparative study of the kinetics of BSA adsorption on the initial and modified films was carried out at 25 °C and physiological pH (PBS, pH 7.4). The data obtained, presented in Figure 10A, demonstrate a relatively high speed of the process. The systems reach the equilibrium plateau within 150 min. The measurement of BSA adsorption after 24 h showed a larger amount of protein attached to the initial PLA surface. The dependences obtained can be explained as follows. The high surface area of hydrogel modified films leads to more intensive interactions of proteins with the surface of such matrices within the first few minutes of their contact. At longer interaction times, more hydrophobic PLA films attach a greater amount of protein due to the irreversible hydrophobic forces [44] that, in turn, change the conformation of BSA on the surface, driving out the hydrophobic portions of the molecule. This leads to the attachment of the next BSA molecules via the same hydrophobic interactions.

This supposition is likely to be true if the BSA adsorption isotherms are analyzed (Figure 10B). To build the isotherms, protein concentration in a supernatant was measured after 24 h, e.g., after the time sufficient for the system to reach equilibrium. The initial PLA film exhibits the layer-by-layer adsorption behavior. At the same time, PLA-PAAm and PLA-Alg modified films possessed lower capacities towards the chosen protein, but no equilibrium plateau was revealed. The isotherm curves plotted for the films modified with a hydrogel layer were nearly linear. Thus, the adsorption process does no reach an equilibrium plateau, which corresponds to the maximum surface capacity, with the concentrations under study. The obtained curves could not be described by the Langmuir adsorption isotherm equation. This type of protein interaction with the surface is likely to be caused by the penetration of a protein inside the pores of a gel with a very high inner surface area. Thus, it can be concluded that the modification of PLA films with hydrogels changes their interaction with albumins, which is known to greatly affect the cell-material interaction. No albumin layer on the surface is formed on the modified films due to its absorption by the hydrogel.

The experiment was performed in 0.01 M sodium borate buffer, pH 7.4, at 25 °C with slight stirring at 300 rpm. In the case of kinetic studies, the concentration of the initial BSA solution was 1 mg/mL.

### 3.6. Protein Release from Hydrogel Layers

Many practical applications of biomedical polymers, such as scaffolds for tissue engineering and drug delivery systems, imply the use of different protein or (poly)peptide molecules to amplify the material interaction with cells. For example, scaffolds for bone tissue engineering need to deliver special growth factors [45]. To study the possibility of the application of the described hydrogel layers as the vehicles of continuous protein delivery, the release of model protein (BSA) was studied.

The hydrogels were loaded with 5 mg of BSA by two different methods. In the case of PAAm gel, the protein was added to the polymerization mixture. To load the protein inside Alg gels, they were first swollen at alkaline pH and kept with the solution of BSA for 1 day. The whole protein capture by the gel was proven by the absence of the protein in a supernatant using the Lowry test.

**Figure 11:**
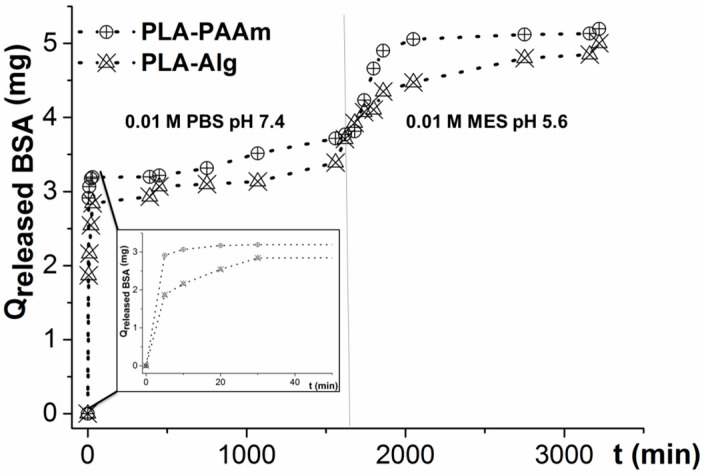
Kinetic curves of BSA release from PAAm and Alg hydrogels on the surface of PLA films. The experiment was performed at pH levels indicated in the figure, at 30 °C with slight stirring (400 rpm).

The release of BSA was studied by immersing the modified films into 0.01 M PBS, pH 7.4 (Figure 11). During the first 20 min, the release was very fast, and was caused by the protein desorption from outer surface of gel. Then the release became slower since the diffusion of a protein in the gel pores needed a much longer time. The release at pH 7.4 finished after approximately one day of experimentation. From our previous studies, it was known that no further release of a protein occurred at such pH. To release the rest of the protein, the buffer was changed from PBS (pH 7.4) to MES (pH 5.6) (Figure 11), in order to change the BSA size and its interaction with the gel. Near total amounts of the protein were released from both PAAm and Alg gels at such conditions. The release from the Alg gel with MES buffer was slightly slower than that observed for PAAm. This fact can be related to the Alg gel compaction leading to the slower BSA diffusion inside the gel. It can be concluded that the release at acidic conditions is generally governed not by gel swelling, but by the change of the protein properties and its interaction with the gel.

### 3.7. Gel Modification with Model Bioligand

The modification of the gels with a model low molecular weight bioligand—glycine—was performed to prove the gels’ potential towards further surface biofunctionalization. In doing so, the surface concentration of reactive carboxylic groups was estimated. The Alg gels contained a large amount of carboxylic groups, which could be easily converted into active ethers. The PAAm gels did not contain the reactive groups. In order to introduce COOH groups, the amide groups were partially hydrolyzed by alkaline. Then, carboxylic groups in both gels were activated by EDC/HOBt and put into reaction with an excess of glycine. The amount of coupled amino acid was determined by the Ninhydrin reaction. As a result, the coupling of low molecular weight ligands was proven and the amount of accessible carboxylic groups was determined.

The results obtained (Table 3) proved the significant capacity of the gel films for the coupling of amino-bearing biomolecules. The amount of coupled amino acid seemed to be equal to the amount of the carboxylic groups on the surface. The number of carboxylic groups was one order of magnitude higher in the case of modified gels compared to the non-modified films. Moreover, the amount of COOH groups in Alg was evaluated to be higher in comparison with the hydrolyzed PAAm gel.

### 3.8. Cell Culture Experiments

**Figure 12:**
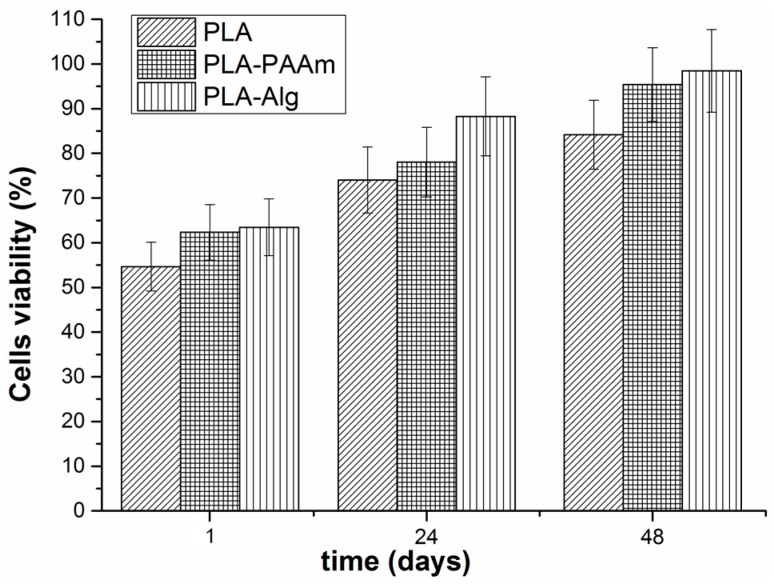
3-(4,5-dimethylthiazol-2-yl)-2,5-diphenyltetrazolium bromide (MTT) assay: cell viability on the surface of the initial PLA and modified PLA-PAAm and PLA-Alg films.

To test the possibility of application of the hydrogel-coated films in tissue engineering, the cell viability on the surface of PLA-PAAm and PLA-Alg was evaluated. The HELF cell line was used for this purpose. The cells were seeded onto the film surface and their viability was determined colorimetrically after 1 and 24 h using a MTT assay. One can observe (Figure 12) that the cell viability on the films with hydrogel layers was higher than that found for the initial PLA film. The PLA-Alg showed maximum cell viability within 24 and 48 h after fibroblast seeding. It seems that this type of hydrogel layer on the surface of PLA-based film is the most favourable for cells attachment and growth. This is in good accordance with previously published results on cell cultivation inside the alginate capsules [46]. The higher cell viability in the case of PLA-Alg could also be the result of the larger surface area in this sample in comparison with the other investigated films (see Table 2).

**Figure 13:**
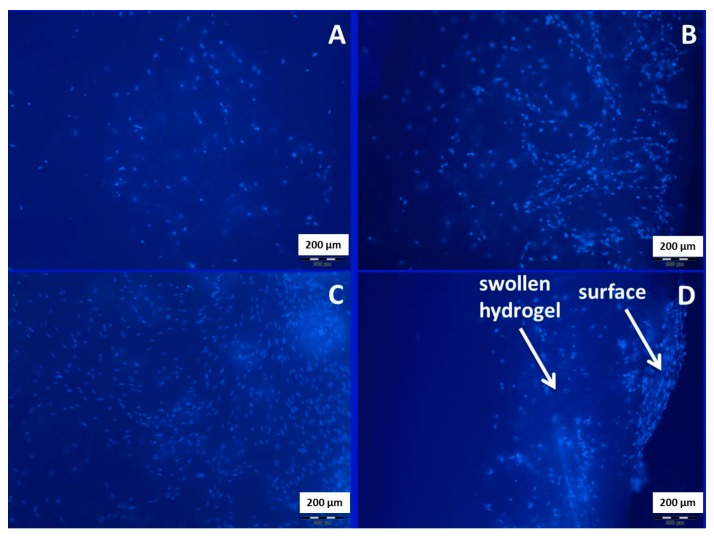
Microphotographs of 4′,6-diamidino-2-phenylindole (DAPI)-stained MCS cells on the surface: (**A**) PLA; (**B**) PLA-PAAm; (**C**) PLA-Alg; (**D**) the PLA-Alg film cut.

Additionally, the HMSC cells were seeded on the film surfaces, cultivated for 3 days, and stained by DAPI in order to visualize cell attachment (Figure 13A–C) and ingrowth (Figure 13D). A greater amount of attached cells was detected in the case of modified PLA films. In this experiment, no visible difference between PLA-PAAm and PLA-Alg gels was found (Figure 13B,C). However, PLA-Alg gel provided the ingrowth of HMCS cells inside the gel.

## 4. Conclusions

The modification of the surface of PLA-based films with covalently attached PAAm and Alg hydrogels was performed. The hydrogel layer thickness affected the surface morphology, hydrophilicity, and swelling, which can be controlled by the monomer/polymer concentration used for modification. The covering of the PLA surface with hydrogel layers was shown to alter its interaction with albumin, as well as its capacity towards the coupling of low molecular weight ligands. The possibility of the application of such hydrogel layers as protein controlled release vehicles was demonstrated. Finally, the enhanced cytocompatibility of modified PLA-based films as compared to the initial PLA layer was proven.

Further studies will be devoted to the molecular imprinting of special biomolecules into the developed hydrogel layers, in order to direct cell-material interactions without the immobilization of expensive molecules. The first experiments on obtaining PAAm and Alg layers imprinted with chymotrypsin have shown that the capacity of molecular-imprinted layers towards this protein is about two times higher than for non-imprinted ones.

## Figures and Tables

**Table 1:** The thickness of dry films and their swelling depending on initial monomer/polymer concentration.

**Initial PLA film**					
Sample	Polymer concentration, wt %		Initial film thickness, µm		Swelling, %
1	5.0		108 ± 32		4 ± 0.6
**PLA film with PAAm gel**					
Sample	Monomers concentration, wt %	[AAm]/[MBAAm], mol/mol	Entire film thickness, µm	Gel layer thickness, µm	Swelling, %
2	1.0	6	122 ± 37	12 ± 4	28 ± 4.2
3	3.0	6	135 ± 41	25 ± 8	30 ± 4.5
4	5.0	6	147 ± 44	34 ± 10	33 ± 5.0
5	3.0	4	141 ± 42	29 ± 9	35 ± 5.3
6	3.0	2	129 ± 32	20 ± 6	27 ± 4.1
**PLA film with Alg gel**					
Sample	Alginate concentration, wt %	Cross-linker concentration (lysine), wt %	Entire film thickness, µm	Gel layer thickness, µm	Swelling, %
7	0.10	0.10	132 ± 40	21 ± 6	12 ± 1.8
8	0.25	0.10	143 ± 43	34 ± 10	26 ± 3.9
9	0.50	0.10	162 ± 49	53 ± 16	38 ± 5.7
10	0.25	0.05	138 ± 41	26 ± 8	32 ± 4.8
11	0.25	2.00	149 ± 45	38 ± 11	41 ± 6.2

**Table 2:** The results of *S*_r_*/S*_0_ parameter calculations for the initial and modified films *.

Calculated parameter	Pure PLA	PLA-PAAm	PLA-Alg
*S*_r_, µm^2^	1550	2134	3475
*S*_r_/*S*_0_	62	85	139

* The parameter is used to quantify the roughness of the modified surface. The *S*_0_ is the product of the area under measurement (5 µm × 5 µm = 25 µm^2^). *S*_r_ was estimated by using software (see Materials and methods section).

**Table 3:** Quantity of glycine coupled to the initial and modified films.

Sample	*q*_init_(Gly), (mg)	*q*_fin_(Gly), (mg)	*q*_coupled_(Gly), (mg)	*q*(–COOH), (μmol)
PLA	0.33	0.27	0.06	0.80
PLA-PAAm	0.33	0.18	0.15	2.00
PLA-Alg	0.33	0.09	0.24	3.24

## Abbreviations:

AAm	acrylamide
Alg	sodium alginate
APS	ammonium peroxodisulfate
BBS	borate buffered solution
BSA	bovine serum albumin
EDC	1-ethyl-3-(3-dimethylaminopropyl)carbodiimide
EDA	ethylene diamine
GMA	glycidyl methacrylate
HOBT	1-hydroxybenzotriazol
HELF	Human embryonic lung fibroblasts
HMSCs	Human mesenchymal stem cells
MBAAm	*N*,*N*′-methylene-bis-acrylamide
PLA	poly(lactic acid)
PLLA	poly(l-lactic acid)
PDLLA	poly(d,l-lactic acid)
PLA-PAAm	PLA-based film the surface of which is modified by PAAm
PLA-Alg	PLA-based film the surface of which is modified by Alg
SDS	sodium dodecyl sulfate
TNBS	2,4,6-trinitrobenzenesulfonic acid
PBS	phosphate buffered solution
